# Metal Complexes of New Bioactive Pyrazolone Phenylhydrazones; Crystal Structure of 4-Acetyl-3-methyl-1-phenyl-2-pyrazoline-5-one phenylhydrazone Ampp-Ph

**DOI:** 10.3390/ijms17050687

**Published:** 2016-05-18

**Authors:** Omoruyi G. Idemudia, Alexander P. Sadimenko, Eric C. Hosten

**Affiliations:** 1 Chemistry Department, University of Fort Hare, Private Bag X1314, Alice 5700, South Africa; asadimenko@ufh.ac.za; 2 Chemistry Department, Nelson Mandela Metropolitan University, Summerstrand Campus, P.O Box 77000, Port Elizabeth 6031, South Africa; eric.hosten@nmmu.ac.za

**Keywords:** phenylhydrazide, dinitrophenylhydrazone, acylpyrazolone, transition metal complexes, TG-DTG, X-ray crystallography, biological studies

## Abstract

The condensation reaction of phenylhydrazine and dinitrophenylhydrazine with 4-acetyl and 4-benzoyl pyrazolone precipitated air-stable acetyldinitrophenylhydrazone Ampp-Dh, benzoylphenylhydrazone Bmpp-Ph and benzoyldinitrophenylhydrazone Bmpp-Dh in their keto imine form; a study inspired by the burning interest for the development of new bioactive materials with novel properties that may become alternative therapeutic agents. Elemental analysis, FTIR, ^1^H, and ^13^C NMR, and mass spectroscopy have been used to justify their proposed chemical structures, which were in agreement with the single crystal structure of Bmpp-Dh earlier reported according to X-ray crystallography. The single crystal structure of 4-acetyl-3-methyl-1-phenyl--pyrazoline-5-one phenylhydrazone Ampp-Ph, which crystallizes in a triclinic crystal system with a P-1 (No. 2) space group is presented. Octahedral Mn(II), Ni(II), Co(II), and Cu(II) complexes of these respective ligands with two molecules each of the bidentate Schiff base, coordinating to the metal ion through the azomethine nitrogen C=N and the keto oxygen C=O, which were afforded by the reaction of aqueous solutions of the corresponding metal salts with the ligands are also reported. Their identity and proposed structures were according to elemental analysis, FTIR spectroscopy, UV-VIS spectrophotometry (electronic spectra) and Bohr magnetic moments, as well as thermogravimetric analysis (TGA) results. A look at the antibacterial and antioxidant activities of synthesized compounds using the methods of the disc diffusion against some selected bacterial isolates and 1,1-diphenyl-2-picryl-hydrazil (DPPH) respectively, showed biological activities in relation to employed standard medicinal drugs.

## 1. Introduction

**Figure 1:**
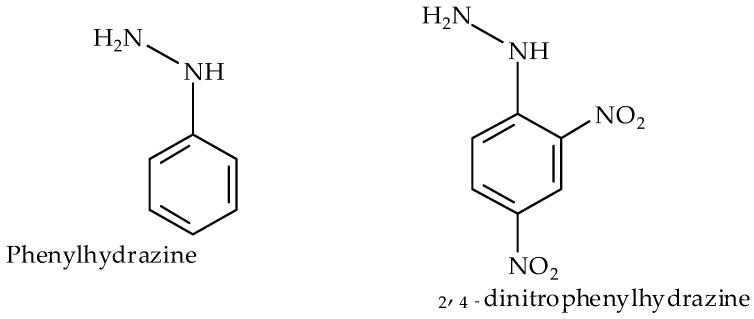
Phenylhydrazine derivatives.

Schiff bases can be said to be one of the most researched group of chemical molecules by scientists, the reasons being their versatility, selectivity, sensitivity, stability, and ease of synthesis, just to mention but a few, which have resulted in their wide applications [1,2,3]. In the midst of their well-researched bioactive medicinal and pharmacological applications [4,5,6], azomethines, as they are also called, either alone or by some sort of modification, have attracted uses as analytical reagents [7], catalysts [8,9], and as azo compounds for use as dyes and pigments [10]. They have also exhibited corrosion inhibition characters for mild steel in H_2_SO_4_ carried out at different temperatures and concentrations [11]; a property that have been attributed to their spatial arrangement and electronic structure [12,13]. It has been reported that the azomethine functional group which they possess contribute to their bioactivity [14], the azomethine nitrogen C=N may interact and form intramolecular hydrogen bonding with some responding sites within the cell structure, which thus affects the regular cell processes; hence, their biological significance [15,16]. Similarly, the lone pair electron in the sp2 hybridized orbital of the azomethine nitrogen is another reason for their chemical reactivity. To a great extent the properties of the Schiff base metal complexes depend on the nature of the ligand and that of the metal ion; usually the Schiff base exhibits a certain electronic environment around a metal ion; therefore, their unique characteristics. 4-acylpyrazolones are di-ketone derivatives of pyrazolone, substituted at the position 4 of the pyrazole ring. Their tautomerism, an ability to exist either in enol or keto structural forms gives them the potential to form different types of interesting coordination compounds [17,18]. Due to their significant pharmacological and biological applications, acylyrazolones have become a more important class of heterocycles [19,20], although their prototypes antipyrines have been synthesized and used as clinical drugs. They serve as useful precursors for the synthesis of superior and more chelating Schiff bases [21,22]. Dinitrophenylhydrazine is a nitro-substituted phenylhydrazine at positions 2 and 4 of its phenyl with the formula C_6_H_6_N_4_O_4_. It is a reddish yellow compound (Figure 1), as reported by Brady and Elsmie, used as an analytical reagent for the quantitative identification of carbonyl functional groups [23]; a property that qualifies it as a good nucleophile for carbonyl aldehydes and ketones in the formation of Schiff bases. We have reported the single crystal structure of the first acylpyrazolone derivative of 2, 4 dinitrophenylhydrazone Bmpp-Dh [24] and that of phenylhydrazone Bmpp-Ph [21], and in continuation of our studies on the design of new metal ion chelating Schiff bases with new chemical properties, presented herein is the synthesis, spectroscopy, thermal, and biological studies of some new acylpyrazolone-based phenylhydrazones and their metal complexes.

## 2. Results

### 2.1. Synthesis

**Figure 2:**
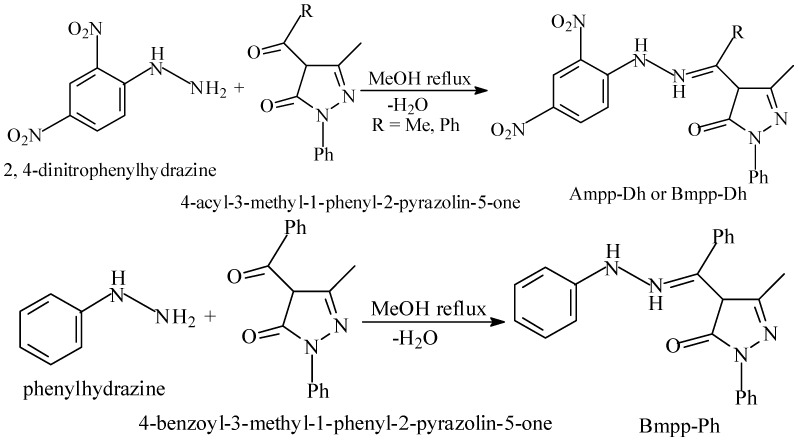
Synthesis scheme of phenylhydrazones.

Acetyl and benzoyl pyrazolone Schiff base precursors were reacted with 2,4-dinitrophenylhydrazine and then with phenylhydrazine in two different one-pot synthesis setups, to afford the Schiff bases; 4-acetyl-3-methyl-1-phenyl-2-pyrazolin-5-one dinitrophenylhydrazone Ampp-Dh, 4-benzoyl-3-methyl-1-phenyl-2-pyrazolin-5-one dinitrophenylhydrazone Bmpp-Dh, and 4-benzoyl-3-methyl-1-phenyl-2-pyrazolin-5-one phenylhydrazone Bmpp-Ph, as shown in the synthesis scheme in Figure 2. We have previously reported the synthesis, characterization, and biological studies of 4-acetyl-3-methyl-1-phenyl-2-pyrazolin-5-one phenylhydrazone Ampp-Ph, as well as a suitable synthesis scheme [25].

**Figure 3:**
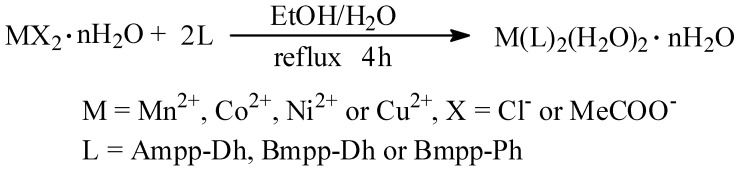
Synthesis scheme of phenylhydrazones metal complexes.

The synthesized Schiff bases were in solid form and stable at room temperature. They are soluble in methanol, ethanol, and most common organic solvents. Schiff bases were precipitated in good yield and purity was confirmed by thin layer chromatography (TLC), which was corroborated by a small melting point range. A total of fourteen metal complexes were synthesized by the treatment of Ampp-Dh, Bmpp-Dh, Ampp-Ph, and Bmpp-Ph with the appropriate metal salt, presented in the synthesis scheme illustrated with an equation in Figure 3. The complexes are generally insoluble in water, ethanol, and in non-coordinating solvents. However, they were soluble in polar solvents with strong donor strength, like Dimethylformamide (DMF) and DMSO. Molar conductance values of metal complexes infer non-electrolyte behavior in DMF [26].

The percentage composition of CHN elemental analysis in Ampp-Dh, Bmpp-Dh, Bmpp-Ph and their metal complexes found, were in agreement with calculated values (refer to the experimental section). Octahedral metal complexes of the bidentate Schiff bases have been proposed. The complexes have two molecules of the bidentate Schiff base and two water molecules each to complete their octahedral geometry [27,28]. Some of their physical properties, melting point range, elemental composition, conductivity, and percentage yield are presented in the experimental section.

### 2.2. ^1^H and ^13^C NMR Spectroscopy

**Figure 4:**
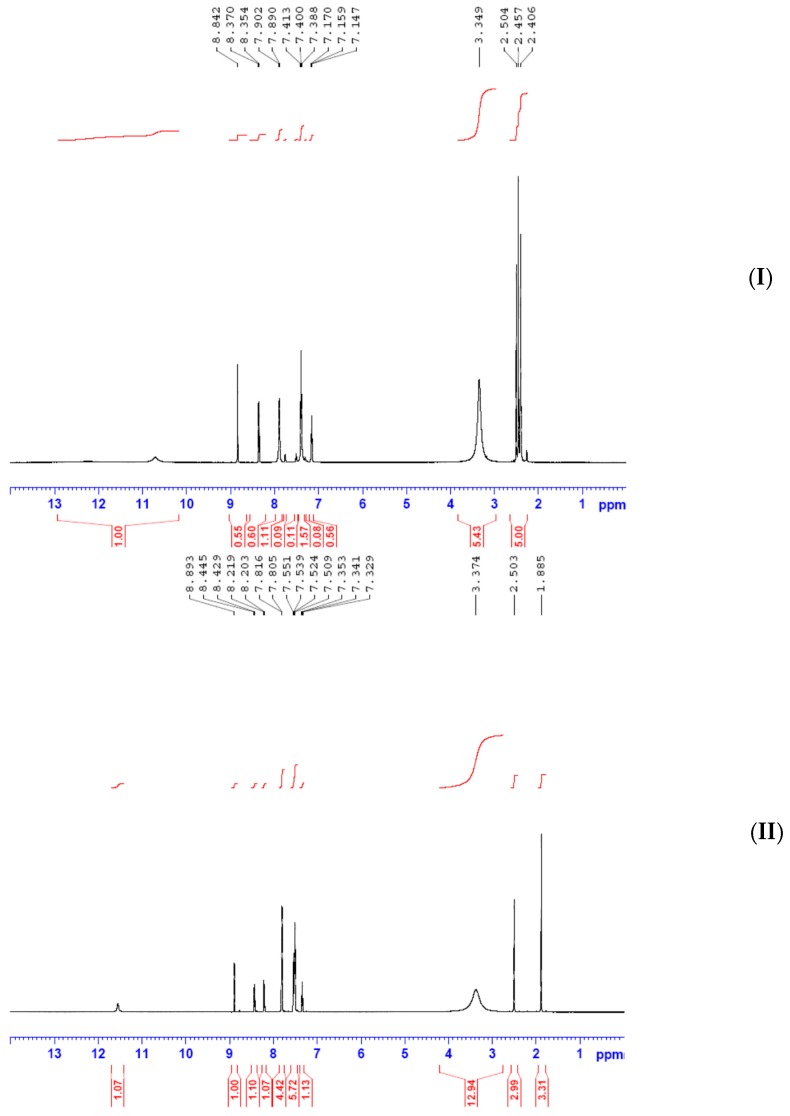
(**I**) ^1^H NMR spectrum of Ampp-Dh; and (**II**) ^1^H NMR spectrum of Bmpp-Dh.

^1^H and ^13^C NMR spectroscopy of synthesized ligands in deuterated DMSO were carefully carried out, with trimethylsilane TMS as the internal standard in DMSO-*d*_6_. Ampp-Dh (Figure 4) showed a singlet with a broad base integrating for one proton at 10.8 ppm which is assigned to the –NH of the dinitrophenylhydrazine. This was followed by another singlet resonating at 8.8 ppm which is due to hydrogen attached to the azomethine carbon atom C=NH. There is a weak signal at 2.4 ppm which may be assigned to the methyl protons but its integration showed otherwise, the reason being that it has probably been unusually merged with the DMSO peak. However, the mass spectrum molecular peak and fragmentations, discussed in Section 2.3 below, corroborates the proposed composition and structure of Ampp-Dh in agreement with the reported crystal structure of a similar compound. The aromatic protons H–Ar are observed as a multiplet at 8.4–7.2 ppm.

**Figure 5:**
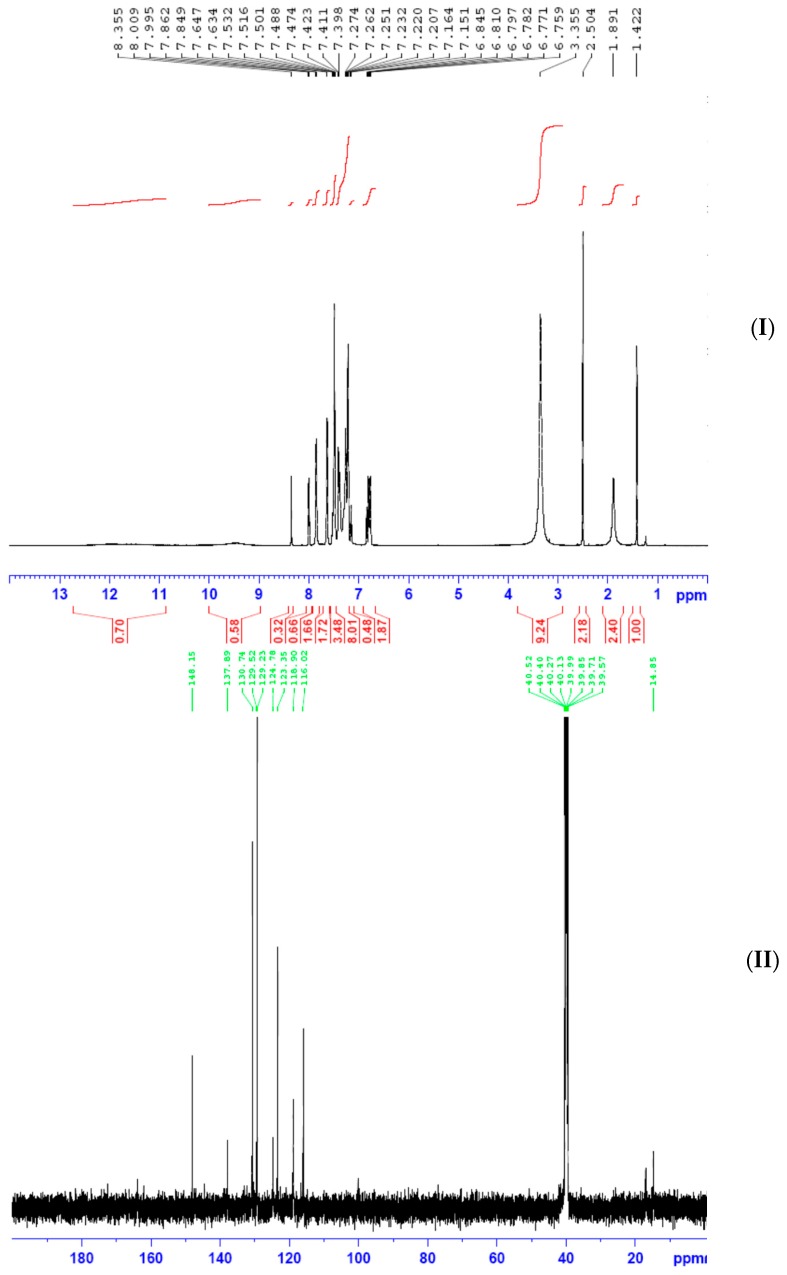
(**I**) ^1^H NMR spectrum of Bmpp-Ph; and (**II**) ^13^C NMR spectrum of Ampp-Dh.

In Bmpp-Dh, the –NH proton and that of the azomethine carbon atom C=NH, respectively, resonated in the downfield region with chemical shift at 11.6 and 8.9 ppm integrating for one proton each. The multiplet, due to the aromatic hydrogens, was observed at 8.5–7.3 ppm, but the integrating protons are not equivalent, which may be due to the overlap of equivalent protons. Finally, the resonance peak due to methyl hydrogens was displayed as a sharp signal upfield of the NMR spectrum at 1.9 ppm integrated for three hydrogen atoms as expected [28]. The ^1^H NMR spectroscopy of Bmmp-Ph (Figure 5) showed similar trend as Bmpp-Dh. The aromatic protons H–Ar were observed as a multiplet at 6.8–8.4 ppm and the resonating peak in the aliphatic region with a chemical shift of 1.9 ppm is due to the methyl protons attached to the pyrazolone ring integrating for approximately three protons. Two broad peaks resonating downfield at around 12.2 and 9.5 ppm integrating for approximately one proton each are assigned to the hydrogen atoms of the –NH and the C=NH azomethine group, respectively.

**Figure 6:**
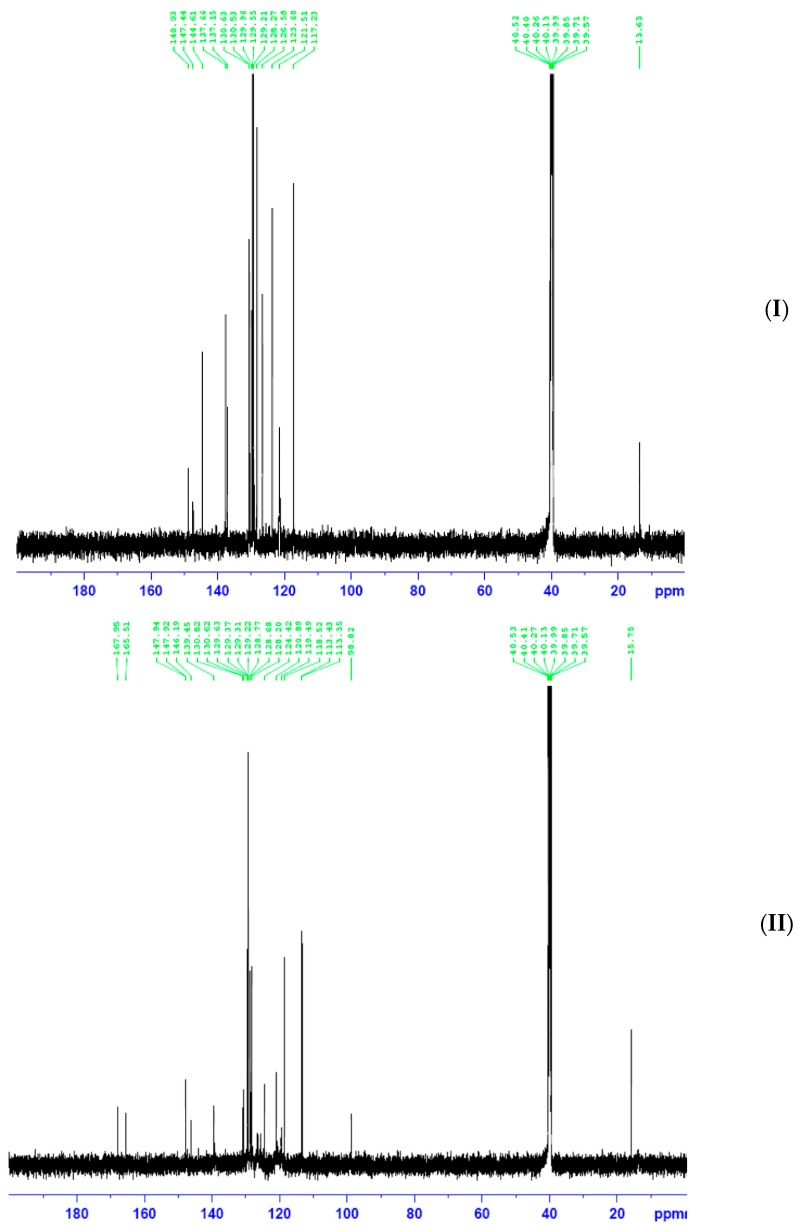
(**I**) ^13^C NMR spectrum of Bmpp-Dh; and (**II**) ^13^C NMR spectrum of Bmpp-Ph.

^13^C NMR spectra of Ampp-Dh, Bmpp-Dh, and Bmpp-Ph displayed aromatic carbon atoms at a chemical shift of 137.9–116.02, 144.6–117.2, and 148.0–115.3 ppm, respectively. Signals due to azomethine carbon C=N and the pyrazolone carbonyl carbon C=O are observed at 137.7 and at 146.2 ppm, respectively in Ampp-Dh [29]. The two methyl group carbons are observed at 16.2 and 14.9 ppm assigned to the pyrazolone methyl and the acetyl methyl, respectively. In Bmpp-Dh, resonance bands due to C=N and C=O are seen at a chemical shift of 147.44 and 148.9 ppm, respectively (Figure 6). Finally, in Bmpp-Dh, the only methyl carbon appears as a single signal in the aliphatic region at 13.6 ppm.

A signal due to pyrazolone methyl carbon resonates at 15.8 ppm in the Bmpp-Ph ^13^C NMR spectrum and the resonance signals at 168.0 ppm may be assigned to the carbonyl carbon of the pyrazolone C=O. Additionally, the signal at 165.5 ppm is due to the azomethine carbon atom C=N [29] and the pyrazolone methyl carbon signal resonated at 15.8 ppm.

### 2.3. Mass Spectroscopy

**Figure 7:**
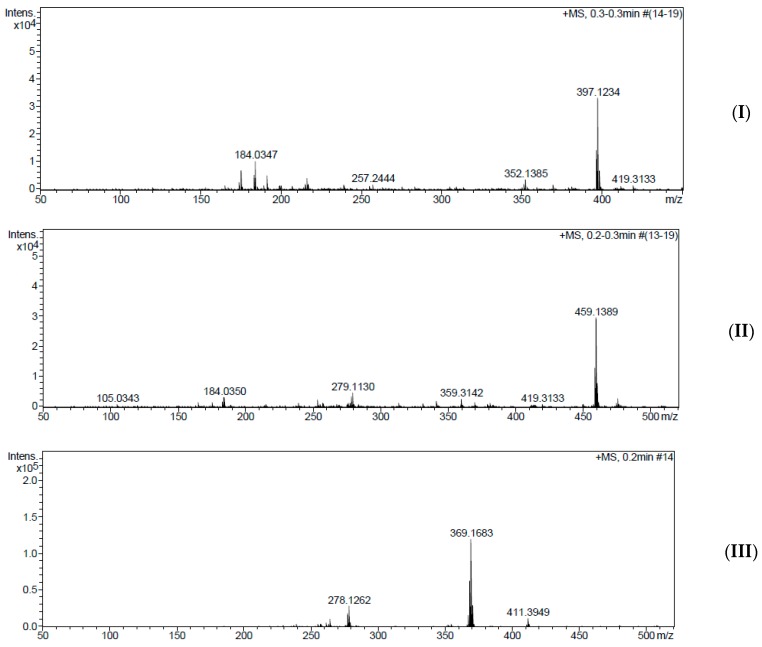
(**I**) Mass spectrum of Ampp-Dh; (**II**) mass spectrum of Bmpp-Dh; and (**III**) mass spectrum of Bmpp-Ph.

The prominent peaks at 100% observed in the mass spectra of Ampp-Dh, Bmpp-Dh, and Bmpp-Ph correspond to the molecular ion M^+^ at *m*/*z* 397, *m*/*z* 459, and *m*/*z* 369, respectively (Figure 7). The molecular ions observed in the Schiff base ligands confirm the calculated theoretical molar mass plus a proton [M + H]^+^.

The peak at *m*/*z* 279 in Bmpp-Dh is due to the protonated benzoyl pyrazolone Schiff base precursor, C_17_H_14_N_2_O_2_ fragmentation, which is observed at *m*/*z* 278 in Bmpp-Ph but for an unprotonated Schiff base precursor.

### 2.4. X-ray Crystallography

The reported Ampp-Ph single crystal structure was obtained from a slow evaporation of its DMF solution. A summary of crystal information is presented in Table 1.

**Figure 8:**
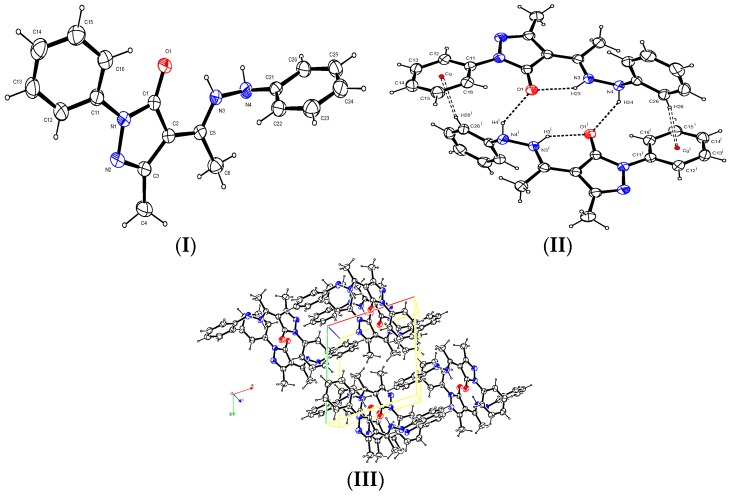
X-ray crystal structures: (**I**) Ortep diagram of the titled compound with thermal ellipsoids drawn at 50% probability level; (**II**) hydrogen interactions; for clarity the π…π ring interaction is not shown. Cg is the centroid of the C11–C16 phenyl group, Symmetry element (i) 1-x, -y, 1-z; and (**III**) packing diagram of Ampp-Ph. The coloured lines are the unit cell axis orientation and the red and blue circles represent oxygen and nitrogen respectively.

In the structure of Ampp-Ph, the phenyl on the dihydropyrazole group is turned slightly out of the dihydropyrazole least square plane by 24.90(5)°. The phenyl on the hydrazine group is turned out more and makes a least square dihedral angle of 84.64(3)° with the dihydropyrazole plane (Figure 8).

There is one short intramolecular hydrogen bond N3–H3…O1 of length 1.931(15) Å which, in terms of graph-set analysis [30,31], necessitates a S^1^_1_(6) descriptor on the unary level. Adjacent molecules have two N4–H4…O1 intermolecular hydrogen bonds of lengths 2.169(15) Å that have a R^2^_2_(14) descriptor on the unary level. The C26–H26 bond has an intermolecular π ring interaction with a C11-C16 phenyl group with a hydrogen to centroid distance of 2.75 Å. The shortest π ring interaction is between adjacent intermolecular C21–C26 phenyl rings with a centroid-to-centroid distance of 3.7827(7) Å and a slippage of 1.474 Å.

### 2.5. Infrared Spectroscopy

The FTIR spectra of the azomethine ligands and their metal complexes were closely compared and the modifications in frequency vibration due to the interaction of the metal ion with the ligands were recorded. Octahedral complexes of the synthesized acylpyrazolone ligands have been proposed, which were in accordance with previously reported [27,28]. Ampp-Dh, Bmpp-Dh, and Bmpp-Ph exist in keto tautomer form, with FTIR spectra having a strong band vibrating at 1627, 1642, and 1634 cm^−1^, respectively, corresponding to the azomethine ν(C=N). In the metal complexes, the metal ion coordinates through the donor nitrogen of the ligands azomethine group and based on this, the azomethine vibration in the FTIR spectra of metals complexes are observed at a lower wavenumber in the range of 1620–1617 cm^−1^ [32]. The broad band at 3486 cm^−1^ in Ampp-Dh, 3489 cm^−1^ in Bmpp-Dh, and at 3491 cm^−1^ in Bmpp-Ph, due to inter and intramolecular hydrogen bonding of the ν(N–H) stretching frequency was observed in the wave number range of 3481–3384 cm^−1^ in the metal complexes, attributed to ν(N–H) and ν(O–H) from the water molecules [33]. The ketone carbonyl ν(C=O) group was observed at 1498, 1501, and 1501 cm^−1^ in Ampp-Dh, Bmpp-Dh, and Bmpp-Ph, respectively, which was absent in the FTIR spectra of the metal complexes by way of forming –C–O–M bonds, an evidence of the coordination of the metal ion through the oxygen of the carbonyl ketone [34]. The two coordinated water molecules that completed the proposed octahedral geometry of metal complexes can be corroborated with the existence of a new band at around 849–830 cm^−1^ [33]. New bands at around 633–620 cm^−1^ and 492–478 cm^−1^ observed in the FTIR spectra of the transition metal complexes were assigned to the newly-formed ν(M–N) and ν(M–O) bonds, respectively [35].

### 2.6. UV-VIS Spectroscopy and Magnetic Moments

The ligands and their metal complexes showed absorption bands in the UV region due to π→π* and n→π* transitions [36]. The absorption bands displayed some modifications in the spectra of metal complexes as a result of metal ion coordination [37]. In general, the electronic spectra of most metal complexes reported exhibited unusual transitions but their magnetic moment values in Bohr Magneton (BM), agreed with theoretical predictions in line with established data. The energy values’ closeness of different states of transitions may be the reason for the disappearance of some expected bands. Electronic spectra absorption bands and values in nanometers are displayed in the experimental section.

**Figure 9:**
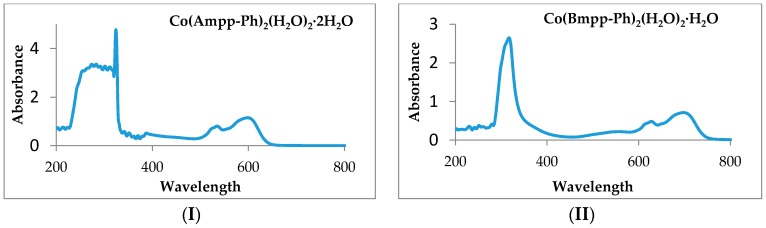
(**I**) Electronic spectra of Co(Ampp-Ph)_2_(H_2_O)_2_∙2H_2_O; and (**II**) electronic spectra of Co(Bmpp-Ph)_2_(H_2_O)_2_∙H_2_O.

Mn(II) complexes of the titled acylpyrazolone-based phenylhydrazones show unusual electronic spectral bands in the visible region but close to the UV region. Two bands were observed at 436 and 462 nm in Mn(Bmpp-Dh)_2_(H_2_O)_2_ and a band each at 451 nm in Mn(Ampp-Dh)_2_(H_2_O)_2_∙H_2_O and 393 nm in Mn(Bmpp-Ph)_2_(H_2_O)_2_∙2H_2_O. These bands may be attributed to ^6^A_1g_→^4^A_1g_ transition a characteristic of an octahedral Mn(II) complex [38]. The magnetic moment of synthesized phenylhydrazone Schiff base complexes with Mn(II), which further justifies their geometry, were 5.65, 5.67, and 5.63 BM for Mn(Bmpp-Dh)_2_(H_2_O)_2_, Mn(Ampp-Dh)_2_(H_2_O)_2_∙H_2_O, and Mn(Bmpp-Ph)_2_(H_2_O)_2_∙2H_2_O, respectively [39]. Co(Bmpp-Dh)_2_(H_2_O)_2_∙H_2_O displayed bands at 425 and 460 nm, and Co(Ampp-Dh)_2_(H_2_O)_2_ showed a band at 414 nm, followed by another one at 493 nm, corresponding to (^4^T_2g_→^4^T_1g_) and (^4^A_2g_→^4^T_1g_) transition of an octahedral Co(II) complex, respectively. Co(Bmpp-Ph)_2_(H_2_O)_2_∙H_2_O and Co(Ampp-Ph)_2_(H_2_O)_2_∙2H_2_O exhibited similar transitions (Figure 9). Co(Bmpp-Ph)_2_(H_2_O)_2_∙H_2_O showed a weak, broad, and almost unnoticeable band at 554 nm, followed by a second band at 627 nm shoulder to a third band at 708 nm. Three bands were also observed for Co(Ampp-Ph)_2_(H_2_O)_2_∙2H_2_O in the visible region of its electronic spectrum at 390, 536, and 600 nm assigned to (^4^T_2g_→^4^T_1g_), (^4^A_2g_→^4^T_1g_) and (^4^T_1g_(p)→^4^T_1g_) transitions, respectively. The measured magnetic moment for all four Co(II) complexes were between 4.45 and 4.48 BM, which were in agreement with the expected values for an octahedral d^7^ Co(II) complex [40].

**Figure 10:**
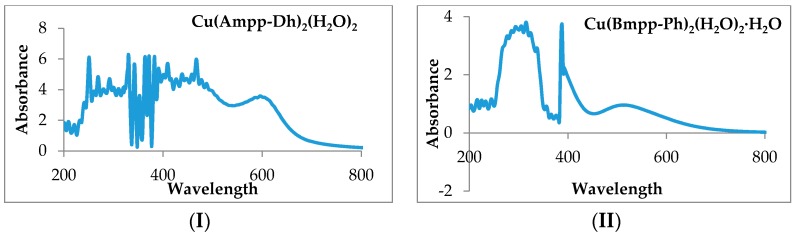
(**I**) Electronic spectra of Cu(Ampp-Dh)_2_(H_2_O)_2_; and (**II**) electronic spectra of Cu(Bmpp-Ph)_2_(H_2_O)_2_∙H_2_O.

The d-d absorption bands in Ni(Bmpp-Dh)_2_(H_2_O)_2_∙H_2_O were observed at 431 and 463 nm, and that of Ni(Ampp-Dh)_2_(H_2_O)_2_ was seen as one band at 436 nm. Two bands in the visible region were also observed in the Ni(Bmpp-Ph)_2_(H_2_O)_2_∙2H_2_O spectrum at 678 and 808 nm. These bands are assigned to ^3^A_2g_→^3^T_1g_(F) and ^3^A_2g_→^3^T_2g_(F) transitions for an octahedral Ni(II) complex. Ni(II) complexes reported herein have a magnetic moment value of between 2.90 and 2.98 BM in further support of the proposed octahedral geometry around the nickel ion in agreement with reported data [41]. Cu(Bmpp-Dh)_2_(H_2_O)_2_∙2H_2_O exhibited two bands at 425 and 460 nm corresponding to ^2^B_1g_→^2^E_g_ transition associated with a magnetic moment of 1.95 BM. Cu(Ampp-Dh)_2_(H_2_O)_2_ on the other hand, having a magnetic moment of 1.97 BM, shows a band at 467 nm attributed to ^2^B_1g_→^2^E_g_ transition, and a broad band peculiar to a distorted octahedral Cu(II) complex at 598 nm corresponding to ^2^B_1g_→^2^B_2g_, Figure 10.

Additionally, a broad band corresponding to a distorted octahedral Cu(II) with a d^9^ configuration at 516 nm assigned to ^2^B_1g_→^2^B_2g_ transition was observed in the Cu(Bmpp-Ph)_2_(H_2_O)_2_∙H_2_O spectrum (Figure 10) and the same transition is seen as a weak, but broad, band at 594 nm for Cu(Ampp-Ph)_2_(H_2_O)_2_ [41]. Cu(Bmpp-Ph)_2_(H_2_O)_2_∙H_2_O had a magnetic moment of 1.94 BM and Cu(Ampp-Ph)_2_(H_2_O)_2_, a magnetic moment of 1.93 BM which corroborates the d^9^ octahedral Cu(II) complex proposed [39].

### 2.7. Thermogravimetric Studies

Metal complexes of phenylhydrazones reported herein are generally thermally stable. In their thermograms, a multistep decomposition pattern is observed with the final decomposition occurring beyond 900 °C, marking their corresponding weight loss assignment unfavorable. However, it is expected that final decomposition will be equivalent to the weight loss associated with residue of the corresponding metal oxides. The two coordinated water molecules common to all metal complexes are expected to decompose at around 140–300 °C [42] and the uncoordinated water usually decomposes a little earlier.

**Figure 11:**
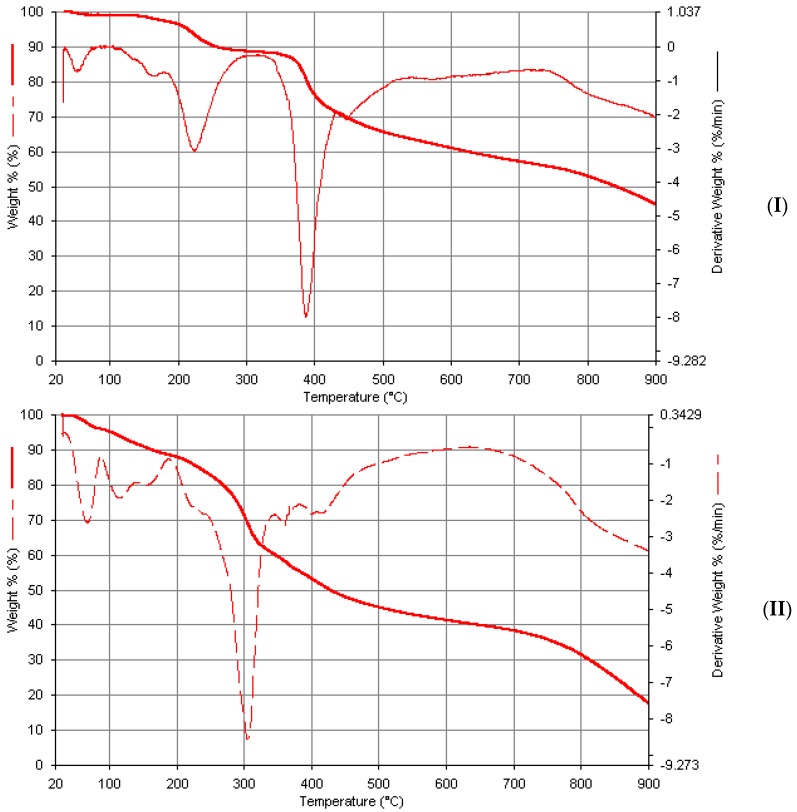
Thermogravimetric analysis curves of metal complexes. (**I**) Mn(Bmpp-Dh)_2_(H_2_O)_2_; and (**II**) Mn(Ampp-Dh)_2_(H_2_O)_2_∙H_2_O.

Two decompositions of about 8% weight loss calculated as 4% was observed in the thermogram of Mn(Bmpp-Dh)_2_(H_2_O)_2_ at 240 °C, associated with the coordinated and uncoordinated water molecules, as well as other water molecules which the complex have absorbed as lattice water (Figure 11). At around 390 °C, a major decomposition occurred which may be due to the removal of one of its ligands calculated as 46%, and followed by another one over a wide temperature range extending above 900 °C, which is as a result of the removal of the other Schiff base ligand leaving behind the MnO residue. A similar decomposition pattern was observed in Mn(Ampp-Dh)_2_(H_2_O)_2_∙H_2_O (Figure 11), with a weight loss due to the coordinated and non-coordinated water molecules around 190 °C with a weight percentage loss of 7%, theoretically calculated as 6%. The major decomposition at around 310 °C may be due to one Schiff base ligand calculated for 44%, and the wide temperature range decomposition extending beyond 900 °C, tentatively, due to the second Schiff base ligand molecule leaving behind the manganese oxide.

**Figure 12:**
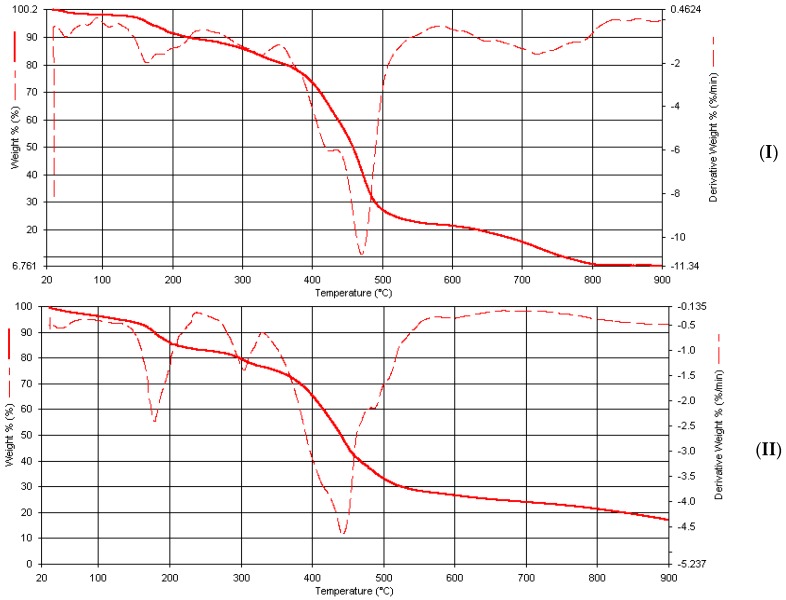
Thermogravimetric analysis curves of metal complexes. (**I**) Mn(Bmpp-Ph)_2_(H_2_O)_2_∙2H_2_O; and (**II**) Co(Bmpp-Ph)_2_(H_2_O)_2_∙H_2_O.

In Mn(Bmpp-Ph)_2_(H_2_O)_2_∙2H_2_O (Figure 12), the first few decompositions observed, totaling to about 8.0% at around 200 °C, are probably due to the removal of four coordinated and uncoordinated water molecules calculated as 8.4%. A major decomposition at 475 °C with a % weight loss of approximately 48% may be due to the removal of one Schiff base molecule, which is theoretically calculated as 43%. A final decomposition at 800°C with a total mass loss of approximately 93% is, tentatively, due to the removal of the water molecules and the Schiff base ligands calculated as approximately 92%, leaving behind the corresponding manganese oxide with approximately 7%, calculated as 8.2%.

The removal of the water molecules can be observed in the thermogram of Co(Bmpp-Dh)_2_(H_2_O)_2_∙H_2_O, as a multiple decomposition between 0 and 240 °C with a total mass percentage loss of about 11% which is calculated as 5%. The major decomposition at 360 °C may be due to one of the Schiff base ligands. Additionally, the last decomposition observed extends beyond 900 °C. The absence of an uncoordinated water molecule is evident in the thermogram of Co(Ampp-Dh)_2_(H_2_O)_2_. The major decomposition at 370 °C may be due to the elimination of the two coordinated water molecules. The other two decompositions observed from Thermogravimetric derivative (DTG) at around 510 and 600 °C could not be assigned. The formation of a gaseous product may be the cause of the unusual thermograms observed.

**Figure 13:**
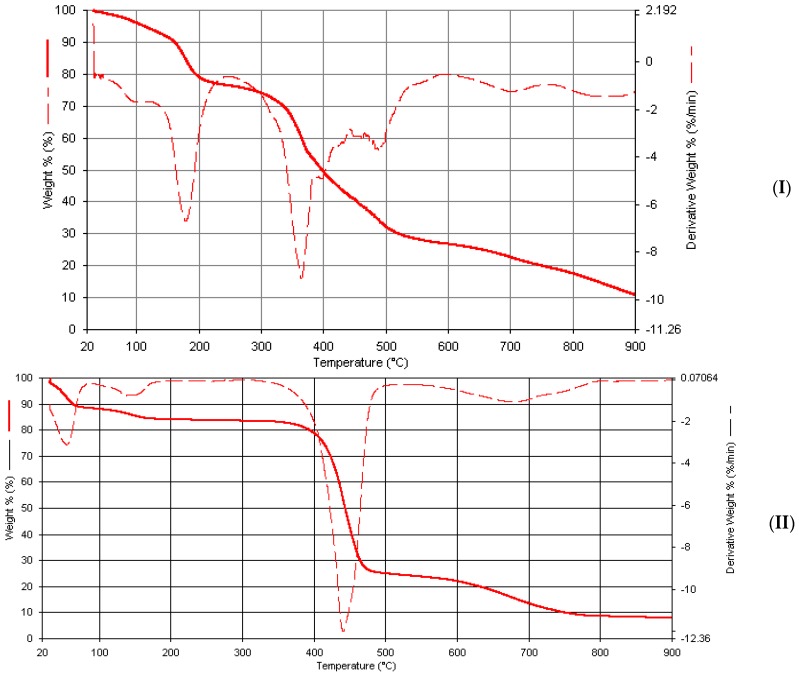
Thermogravimetric analysis curves of metal complexes. (**I**) Co(Ampp-Ph)_2_(H_2_O)_2_∙2H_2_O; and (**II**) Ni(Bmpp-Ph)_2_(H_2_O)_2_∙2H_2_O.

The thermal analysis of both Co(Bmpp-Ph)_2_(H_2_O)_2_∙H_2_O and Co(Ampp-Ph)_2_(H_2_O)_2_∙2H_2_O, as seen in their thermograms, exhibited multistep decompositions. However, the decomposition around 170 and 300 °C corresponds to the elimination of water molecules. The final weight loss of 92.3%, calculated as 91.2% at >900 °C for Co(Bmpp-Ph)_2_(H_2_O)_2_∙H_2_O, and a final weight loss of 89.9%, calculated as 90.6% at >900 °C for Co(Ampp-Ph)_2_(H_2_O)_2_∙2H_2_O (Figure 13), corresponding to the decomposition of the water molecules and ligands, respectively, were observed, leaving behind a residue of cobalt oxide with percentage mass of 10.4% (calc. 9.5%) and 10% (calc. 10.1%), respectively.

Removal of the three water molecules in Ni(Bmpp-Dh)_2_(H_2_O)_2_∙H_2_O was evident from the multistep decomposition with 8% weight loss, theoretically calculated as 7%, occurring at around 240 °C. This was followed by a major decomposition at 390 °C which may be assigned to the removal of one Schiff base ligand. The last decomposition over a wide range of temperature may be due to the second Schiff base leaving behind the residue of the metal oxide.

A similar pattern was observed in Ni(Ampp-Dh)_2_(H_2_O)_2_. The water molecule removal was evident from the DTG curve of decomposition at 160 °C, and the major decomposition at around 298 °C may be attributed to the removal of the ligand, although the decomposition temperature is low. Multiple decompositions are observed in Ni(Ampp-Dh)_2_(H_2_O)_2_ and, as such, the metal complex component assignments were not possible at this time.

The total weight percent loss of about 12% in two steps observed in the Ni(Bmpp-Ph)_2_(H_2_O)_2_∙2H_2_O thermogram can be attributed to the loss of water molecules (Figure 13). A major decomposition is observed at around 440 °C with a mass percentage loss of 58%, which may be attributed to one molecule of the ligand, calculated at approximately 43%. The third decomposition at 760 °C may be the removal of the second ligand molecule, leaving behind a residue of the metal oxide with a weight percentage loss of 8% (calc. as 8.6%) in Ni(Bmpp-Ph)_2_(H_2_O)_2_∙2H_2_O.

Both copper complexes of the two 2,4-dinitrophenylhdrazones exhibited a major decomposition at 380 °C, this decompositions may be due to the removal of one Schiff base ligand, followed by another decomposition over a wide temperature range which is associated with the removal of the second Schiff base. The presence of the uncoordinated water molecule is obvious in Cu(Bmpp-Dh)_2_(H_2_O)_2_∙2H_2_O with a decomposition at around 100 °C, followed by the removal of a coordinated water molecule at 200 °C. The DTG curve reveals some decomposition around 220 °C which is due to the coordinated water molecule and absence of any decomposition earlier than this is evidence of no lattice water molecules.

**Figure 14:**
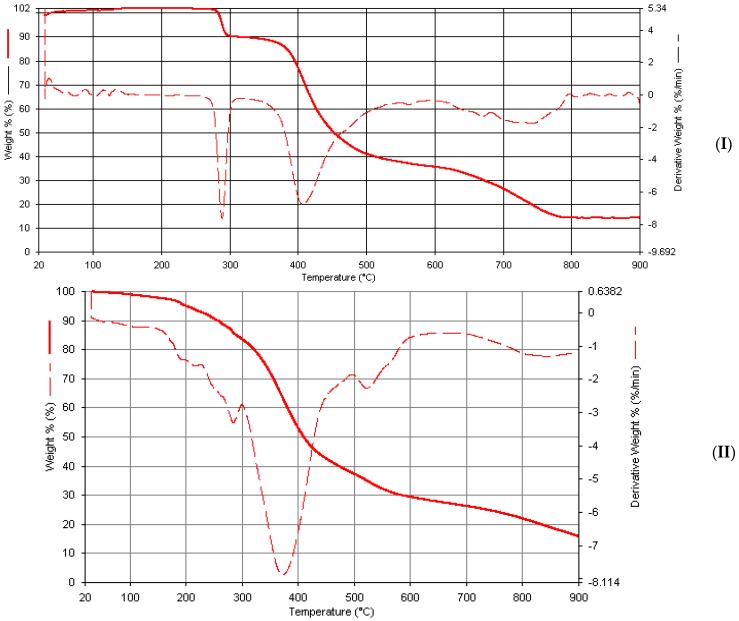
Thermogravimetric analysis curves of metal complexes. (**I**) Cu(Bmpp-Ph)_2_(H_2_O)_2_∙H_2_O; and (**II**) Cu(Ampp-Ph)_2_(H_2_O)_2_.

Three main decompositions has been observed for Cu(Bmpp-Ph)_2_(H_2_O)_2_∙H_2_O amongst other decompositions according to the DTG curve that could not be directly assigned (Figure 14). However, the decomposition at 290 °C may be as a result of the removal of the two coordinating water molecules, followed by a second major mass percentage loss at 420 °C assigned to the removal of a molecule of the Schiff base ligand. The final mass percentage loss at 785 °C is equivalent to the removal of the water molecules and the ligands, leaving behind the copper oxide with a mass percentage of approximately 12%, which is calculated as 10%. One major decomposition at 370 °C was obtained in the thermogram of Cu(Ampp-Dh)_2_(H_2_O)_2_ (Figure 14), which may be assigned to the removal of the two coordinating water molecules. Although other components of the copper complex cannot be accounted for with the minimal information from its TG/DTG curves, a final decomposition at >900 °C gave a residue of CuO with a percentage mass of 11.8%, calculated for 11.2%.

**Figure 15:**
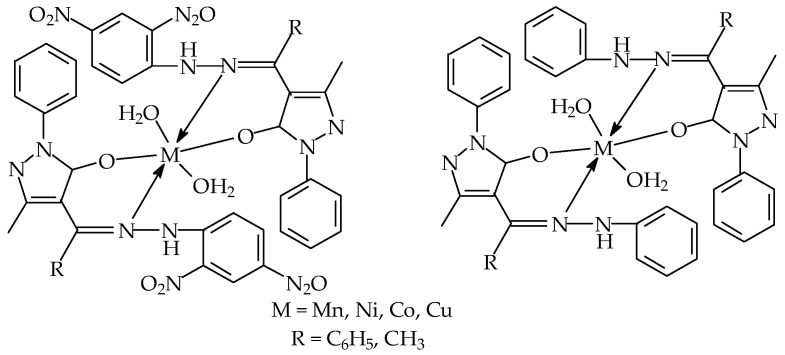
Proposed structure schemes of phenylhydrazone metal complexes.

Based on elemental analysis, spectroscopic and thermal studies, the proposed structural scheme for metal complexes is presented in Figure 15.

### 2.8. Biological Studies

The zones of inhibition from *in vitro* antibacterial screening of phenylhydrazones and their metal complexes are reported, using the paper disc diffusion method, are presented in Table 2. The compounds have shown a generally low antibacterial activity compared to chloramphenicol with different inhibition zones. Ampp-Dh showed a highest zone of inhibition at 24 mm against *Staphylococus aureus*, which was followed by Co(Ampp-Dh)_2_(H_2_O)_2_ with 20 and 15.5 mm against Gram-negative *Aeromonas hydrophillia* and *Staphylococus aureus*, respectively. Ni(Bmpp-Dh)_2_(H_2_O)_2_∙H_2_O displayed a broad spectrum activity, having a positive inhibition against all selected bacterial isolates and may be referred to as most active compound on the list [43].

The antioxidant (free radical scavenging) activity of synthesized compounds under investigation, against DPPH, were of interesting characteristics, although at 0.50 mg/mL the two 2,4-dinitrophenylhydrazones, Mn(Bmpp-Dh)_2_(H_2_O)_2_, Co(Ampp-Dh)_2_(H_2_O)_2_, and Cu(Bmpp-Ph)_2_(H_2_O)_2_∙H_2_O did not show any activity. Cu(Bmpp-Dh)_2_(H_2_O)_2_∙2H_2_O had the strongest antioxidant activity with a highest percentage scavenging activity value of 89.90% at 0.25 mg/mL, close to that of the standard drug, ascorbic acid (Table 3). This was in agreement with reported work on synthesized compounds with metal ion coordination [44].

**Figure 16:**
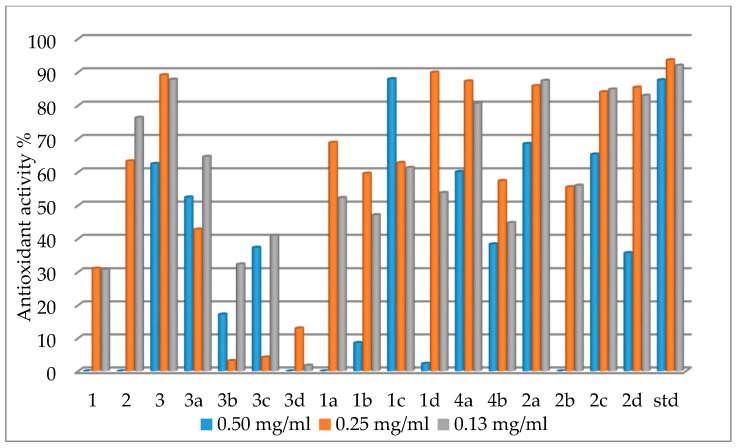
Chart showing the antioxidant activities of phenylhydrazones and their metal complexes.

The metal complexes with Bmpp-Dh exhibited a generally-increased activity than their ligand. Specifically, Ni(Bmpp-Dh)_2_(H_2_O)_2_∙H_2_O and Cu(Bmpp-Dh)_2_(H_2_O)_2_∙2H_2_O showed a significant increase in antioxidant property at 0.5 and 0.25 mg/mL, respectively, compared to their free Schiff base ligand (Figure 16).

Additionally, in Figure 16, a generally higher antioxidant activity for metal complexes have been observed with those of acetylpyrazolone-based 2,4-dinitrophenylhydrazone and this was peculiar in all different concentrations. Mn(Ampp-Dh)_2_(H_2_O)_2_∙H_2_O exhibited the strongest activity across all concentration values.

## 3. Materials and Methods

### 3.1. Materials and Physical Measurements

Commercially available analytical reagents, transition metal salts, dinitrophenylhydrazine, phenylhydrazine, 3-methyl-1-phenyl-2-pyrazolin-5-one, and solvents were used as supplied. The melting point of synthesized compounds that gives, somewhat, of an idea of the molecules’ purity was performed using the GallenKamp melting point apparatus (Northampton, UK). Elemental analyses to determine the compositions in percentage of CHN were carried out on a LECO.TRUSpec Micro CHNS analyzer (St. Joseph, MI, USA). FTIR spectra were measured on a Perkin-Elmer Model System 2000 FTIR spectrometer (Shelton, CT, USA) using KBr pellets (370–4000 cm^−1^). The electronic spectra of metal complexes were made possible using a Perkin-Elmer Lambda 25 spectrometer (Waltham, MA, USA). A Sherwood Scientific magnetic susceptibility balance was used for magnetic moments of the complexes at room temperature and diamagnetism corrections were estimated from Pascal’s constants. ^1^H and ^13^C NMR spectra were recorded in deuterated DMSO on a Bruker 600 MHz Avance II NMR spectrophotometer (Lyon, Rhône-Alpes, France) using trimethylsilane TMS, as the internal standard. Thermal analyses were done on a NETZSCH STA 449 C instrument (Selb, Bavaria, Germany) at a temperature range of 20–900 °C with a heating rate of 20 °C·min^−1^ in nitrogen gas. The Bruker micrOTOF-Q II 10390 mass spectrometer (Billerica, MA, USA) was employed to measure the mass spectra of ligands, which was analyzed with ACPI using a direct insertion probe (DIP). An external calibration with sodium formate was performed to attain the correct accurate mass. Single crystal X-ray diffraction studies were performed on a Bruker Kappa Apex II diffractometer (Madison, WI, USA) with graphite-monochromated Mo Kα radiation (λ = 0.71073 Å). Acylpyrazolone Schiff base precursors 4-acetyl-3-methyl-1-phenyl-2-pyrazolin-5-one and 4-benzoyl-3-methyl-1-phenyl-2-pyrazolin-5-one were synthesized as previously reported [45].

### 3.2. Synthesis of Phenylhydrazones

A solution of 4-acetyl-3-methyl-1-phenyl-2-pyrazolin-5-one (2.0 mmol, 0.43 g) and 4-benzoyl-3-methyl-1-phenyl-2-pyrazolin-5-one (2.0 mmol, 0.56 g) in methanol (40 mL), each in a separate round-bottom flask, was reacted with phenylhydrazine (2.0 mmol, 0.22 g) in methanol (10 mL) and 2,4-dinitrophenylhydrazine (2.0 mmol, 0.40 g) in hot methanol (40 mL) under reflux for 4 h to precipitate red 4-acetyl-3-methyl-1-phenyl-2-pyrazolin-5-one dinitrophenylhydrazone (Ampp-Dh), red 4-benzoyl-3-methyl-1-phenyl-2-pyrazolin-5-one dinitrophenylhydrazone (Bmpp-Dh) and yellow 4-beenzoyl-3-methyl-1-phenyl-2-pyrazolin-5-one phenylhydrazone (Bmpp-Ph). The resulting solids in each case were filtered, washed with methanol, and dried at room temperature. They were recrystallized from methanol and stored over fused CaCl_2_. Gold color Ampp-Ph was synthesized in a similar way as reported earlier [25], and its golden brown block like single crystals suitable for X-ray crystallography were grown from slow evaporation of its DMF solution.

#### 3.2.1. 4-Acetyl-3-methyl-1-phenyl-2-pyrazolin-5-one dinitrophenylhydrazone Ampp-Dh

Yield: 74%. M.p.: 217−218 °C. ^1^H NMR (600 MHz, DMSO): δ (ppm) = 10.8 (br, 1H, phenylhydrazine –NH), 8.8 (s, 1H, C=N–H), 8.4–7.2 (m, 8H, aromatic–H), 2.30 (s, 3H, CH_3_). ^13^C NMR (600 MHz, DMSO): δ (ppm) = 146.2 (s, C=O), 137.7 (s, C=N), 137.9–116.0 (s, aromatic carbons), 16.2 (s, pyrazolone CH_3_), 14.9 (s, acetyl CH_3_). IR (KBr, cm^−1^): 3486 ν(N–H), 2913 ν(C–H), 1627 ν(C=N), 1501 ν(C=O). APCI-MS: *m*/*z* 397.12 ([M + H]^+^, 100%). Anal. Calcd. C_18_H_16_N_6_O_5_ (396.12 g/mol): C, 54.53%; H, 4.07%; N, 21.21%. Found: C, 54.20%; H, 4.11%; N, 20.98%.

#### 3.2.2. 4-Benzoyl-3-methyl-1-phenyl-2-pyrazolin-5-one dinitrophenylhydrazone Bmpp-Dp

Yield: 73%. M.p.: 240−242 °C. ^1^H NMR (600 MHz, DMSO): δ (ppm) = 11.6 (s, 1H, phenylhydrazine –NH), 8.9 (s, 1H, C=N–H), 8.5−7.3 (m, 13H, aromatic–H), 1.9 (s, 3H, CH_3_). ^13^C NMR (600 MHz, DMSO): δ (ppm) = 148.9 (s, C=O), 147.4 (s, C=N), 144.6–117.2 (m, aromatic carbons), 13.6 (s, pyrazolone CH_3_). IR (KBr, cm^−1^): 3489 ν(N–H), 2954 ν(C–H), 1642 ν(C=N), 1501 ν(C=O). APCI-MS: *m*/*z* 459.14 ([M + H]^+^, 100%). Anal. Calcd. C_23_H_18_N_6_O_5_ (458.13 g/mol): C, 60.24%; H, 3.96%; N, 18.34%. Found: C, 60.18%; H, 3.58%; N, 18.40%.

#### 3.2.3. 4-Benzoyl-3-methyl-1-phenyl-2-pyrazolin-5-one phenylhydrazone Bmpp-Ph

Yield: 71%. M.p.: 197−199 °C. ^1^H NMR (600 MHz, DMSO): δ (ppm) = 12.2 (br, 1H, phenylhydrazine –NH), 9.5 (br, 1H, C=N-H), 8.4−6.8 (m, 15H, aromatic-H), 1.9 (s, 3H, CH_3_). ^13^C NMR (600 MHz, DMSO): δ (ppm) = 168.0 (s, C=O), 165.5 (s, C=N), 148−115.3 (m, aromatic carbons), 15.8 (s, pyrazolone CH_3_). IR (KBr, cm^−1^): 3491 ν(N–H), 2919 ν(C–H), 1634 ν(C=N), 1501 ν(C=O). Elemental analysis for C_23_H_20_N_4_O (%): found C 74.61, H 5.65, N 15.14; calculated C 74.97, H 5.47, N 15.21. Mol. mass (g/mol): 368.16. APCI-MS: *m*/*z* 369.17 ([M + H]^+^, 100%).

### 3.3. Synthesis of Phenylhydrazones Metal Complexes

A solutions of Ampp-Dh (2 mmol, 0.79 g), Bmpp-Dh (2 mmol, 0.92 g), Ampp-Ph (2.0 mmol, 0.43 g), and Bmpp-Ph (2 mmol, 0.74 g) in hot ethanol (40 mL), each in a separate round-bottom flask, were reacted with 1 mmol of aqueous solution of corresponding metal salts while stirring under reflux and followed with the addition of NaOH (2 mmol, 0.08 g), to precipitate the metal complexes after 4 h of reflux. The resulting solutions were filtered, and their precipitates washed with ethanol/water (1:1), dried at room temperature, and stored over fused CaCl_2_.

#### 3.3.1. Bis(4-acetyl-3-methyl-1-phenyl-2-pyrazolin-5-one dinitrophenylhydrazone) Diaquamanganesse (Ii) Monohydrate Mn(Ampp-Dh)_2_(H_2_O)_2_∙H_2_O

Yield: 70%. M.p.: 209−211 °C. Molar cond. (10^−3^ M in DMF): 8.09 ohm^−1^ cm^2^ mol^−1^. µ_eff_(B.M.): 5.67. UV-VIS (DMF) λ_max_ nm: 270 (π→π*), 324, (n→π*), 451 (d→d). IR (KBr, cm^−1^): 3489 ν(N–H), 3395 ν(O‒H), 2925 ν(C–H), 1620 ν(C=N), 840 ν(H_2_O), 621 ν(M–N), 485 ν(M–O). Anal. Calcd. C_36_H_36_N_12_O_13_Mn (899.19 g/mol): C 48.04%, H 4.03%, N 18.69%, Found: C 48.57%, H 5.42%, N 18.10%.

#### 3.3.2. Bis(4-acetyl-3-methyl-1-phenyl-2-pyrazolin-5-one dinitrophenylhydrazone) Diaquacobalt(Ii) Co(Ampp-Dh)_2_(H_2_O)_2_

Yield: 71%. M.p.: 199−201 °C. Molar cond. (10^−3^ M in DMF): 11.80 ohm^−1^ cm^2^ mol^−1^. µ_eff_(B.M.): 4.47. UV-VIS (DMF) λ_max_ nm: 268 (π→π*), 324 (n→π*), 414, 493 (d→d). IR (KBr, cm^−1^): 3493 ν(N–H), 3384 ν(O‒H), 2929 ν(C–H), 1618 ν(C=N), 843 ν(H_2_O), 630 ν(M–N), 484 ν(M–O). Anal. Calcd. C_36_H_34_N_12_O_12_Co (885.17 g/mol): C 48.80%, H 3.87%, N 18.98%, Found: C 48.61%, H 3.63%, N 18.79%.

#### 3.3.3. Bis(4-acetyl-3-methyl-1-phenyl-2-pyrazolin-5-one dinitrophenylhydrazone) Diaquanickel(Ii) Ni(Ampp-Dh)_2_(H_2_O)_2_

Yield: 69%. M.p.: 154−156 °C. Molar cond. (10^−3^ M in DMF): 4.40 ohm^−1^ cm^2^ mol^−1^. µ_eff_(B.M.): 2.92. UV-VIS (DMF) λ_max_ nm: 278 (π→π*), 324 (n→π*), 436, (d→d). IR (KBr, cm^−1^): 3495 ν(N–H), 3388 ν(O‒H), 2921 ν(C–H), 1617 ν(C=N), 849 ν(H_2_O), 633 ν(M–N), 486 ν(M–O). Anal. Calcd. C_36_H_40_N_8_O_9_S_2_Ni (884.93 g/mol): C 48.82%, H 3.87%, N 18.99%, Found: C 48.70%, H 3.85%, N 18.74%.

#### 3.3.4. Bis(4-acetyl-3-methyl-1-phenyl-2-pyrazolin-5-one dinitrophenylhydrazone) Diaquacopper(Ii) Cu(Ampp-Dh)_2_(H_2_O)_2_

Yield: 78%. M.p.: 222−223 °C. Molar cond. (10^−3^ M in DMF): 11.80 ohm^−1^ cm^2^ mol^−1^. µ_eff_(B.M.): 1.97. UV-VIS (DMF) λ_max_ nm: 251 (π→π*), 342 (n→π*), 467, 598, (d→d). IR (KBr, cm^−1^): 3498 ν(N–H), 3390 ν(O–H), 2935 ν(C–H), 1619 ν(C=N), 833 ν(H_2_O), 620 ν(M–N), 492 ν(M–O). Anal. Calcd. C_36_H_34_N_12_O_12_Cu (889.79 g/mol): C 48.55%, H 3.85%, N 18.89%, Found: C 48.49%, H 3.96%, N 18.54%.

#### 3.3.5. Bis(4-benzoyl-3-methyl-1-phenyl-2-pyrazolin-5-one dinitrophenylhydrazone) Diaquamanganese (II) Mn(Bmpp-Dh)_2_(H_2_O)_2_

Yield: 76%. M.p.: 240−241 °C. Molar cond. (10^−3^ M in DMF): 11.40 ohm^−1^ cm^2^ mol^−1^. µ_eff_(B.M.): 5.65. UV-VIS (DMF) λ_max_ nm: 280 (π→π*), 324 (n→π*), 436, 462, (d→d). IR (KBr, cm^−1^): 3496 ν(N–H), 3392 ν(O–H), 2923 ν(C–H), 1619 ν(C=N), 833 ν(H_2_O), 629 ν(M–N), 486 ν(M–O). Anal. Calcd. C_46_H_42_N_8_O_8_S_2_Mn (1003.19 g/mol): C 55.02%, H 3.62%, N 16.75%, Found: C 54.78%, H 3.51%, N 16.41%.

#### 3.3.6. Bis(4-benzoyl-3-methyl-1-phenyl-2-pyrazolin-5-one dinitrophenylhydrazone) Diaquacobalt(Ii) Monohydrate Co(Bmpp-Dh)_2_(H_2_O)_2_∙H_2_O

Yield: 72%. M.p.: 248−250 °C. Molar cond. (10^−3^ M in DMF): 10.91 ohm^−1^ cm^2^ mol^−1^. µ_eff_(B.M.): 4.48. UV-VIS (DMF) λ_max_ nm: 271 (π→π*), 323 (n→π*), 425, 460 (d→d). IR (KBr, cm^−1^): 3484 ν(N–H), 3396 ν(O–H), 2926 ν(C–H), 1620 ν(C=N), 832 ν(H_2_O), 622 ν(M–N), 487 ν(M–O). Anal. Calcd. C_46_H_38_N_12_O_13_Co (1025.20 g/mol): C 53.84%, H 3.74%, N 16.39%, Found: C 53.40%, H 3.92%, N 16.22%.

#### 3.3.7. Bis(4-benzoyl-3-methyl-1-phenyl-2-pyrazolin-5-one dinitrophenylhydrazone) Diaquanickel(Ii) Monohydrate Ni(Bmpp-Dh)_2_(H_2_O)_2_∙H_2_O

Yield: 72%. M.p.: 247−249 °C. Molar cond. (10^−3^ M in DMF): 6.34 ohm^−1^ cm^2^ mol^−1^. µ_eff_(B.M.): 2.90. UV-VIS (DMF) λ_max_ nm: 274 (π→π*), 324 (n→π*), 431, 463 (d→d). IR (KBr, cm^−1^): 3494 ν(N–H), 3394 ν(O–H), 2925 ν(C–H), 1619 ν(C=N), 832 ν(H_2_O), 624 ν(M–N), 478 ν(M–O). Anal. Calcd. C_46_H_38_N_12_O_13_Ni (1024.96 g/mol): C 53.86%, H 3.74%, N 16.39%, Found: C 53.68%, H 3.80%, N 16.32%.

#### 3.3.8. Bis(4-benzoyl-3-methyl-1-phenyl-2-pyrazolin-5-one dinitrophenylhydrazone) Diaquacopper(Ii) Dihydrate Cu(Bmpp-Dh)_2_(H_2_O)_2_∙2H_2_O

Yield: 79%. M.p.: 250−251 °C. Molar cond. (10^−3^ M in DMF): 9.96 ohm^−1^ cm^2^ mol^−1^. µ_eff_(B.M.): 1.95. UV-VIS (DMF) λ_max_ nm: 268 (π→π*), 324 (n→π*), 426, 449 (d→d). IR (KBr, cm^−1^): 3493 ν(N–H), 3396 ν(O‒H), 2930 ν(C–H), 1619 ν(C=N), 830 ν(H_2_O), 623 ν(M–N), 487 ν(M–O). Anal. Calcd. C_46_H_40_N_12_O_14_Cu (1047.82 g/mol): C 52.68%, H 3.89%, N 16.04%, Found: C 52.65%, H 3.75%, N 15.83%.

#### 3.3.9. Bis(4-benzoyl-3-methyl-1-phenyl-2-pyrazolin-5-one phenylhydrazone) Diaquamanganese (Ii) Dihydrate Mn(Bmpp-Ph)_2_(H_2_O)_2_∙2H_2_O

Yield: 73%. M.p.: 120−121 °C. Molar cond. (10^−3^ M in DMF): 8.28 ohm^−1^ cm^2^ mol^−1^. µ_eff_(B.M.): 5.63. UV-VIS (DMF) λ_max_ nm: 228 (π→π*), 303 (n→π*), 393 (d→d). IR (KBr, cm^−1^): 3492 ν(N–H), 3397 ν(O–H), 2943 ν(C–H), 1621 ν(C=N), 841 ν(H_2_O), 631 ν(M–N), 486 ν(M–O). Elemental analysis for C_46_H_46_N_8_O_6_Mn (%): found C 64.00, H 5.53, N 13.07; calculated C 64.09, H 5.38, N 13.01, Mol. mass (g/mol): 861.29.

#### 3.3.10. Bis(4-benzoyl-3-methyl-1-phenyl-2-pyrazolin-5-one phenylhydrazone) Diaquacobalt(Ii) Monohydrate Co(Bmpp-Ph)_2_(H_2_O)_2_∙H_2_O

Yield: 72%. M.p.: 125−127 °C. Molar cond. (10^−3^ M in DMF): 10.02 ohm^−1^ cm^2^ mol^−1^. µ_eff_(B.M.): 4.45. UV-VIS (DMF) λ_max_ nm: 230 (π→π*), 317 (n→π*), 554, 627, 708 (d→d). IR (KBr, cm^−1^): 3480 ν(N–H), 3373 ν(O–H), 2949 ν(C–H), 1618 ν(C=N), 826 ν(H_2_O), 641 ν(M–N), 485 ν(M–O). Elemental analysis for C_46_H_44_N_8_O_5_Co (%): found C 65.06, H 4.96, N 13.14; calculated C 65.15, H 5.23, N 13.22, Mol. mass (g/mol): 847.28.

#### 3.3.11. Bis(4-benzoyl-3-methyl-1-phenyl-2-pyrazolin-5-one phenylhydrazone) Diaquanickel(Ii) Dihydrate Ni(Bmpp-Ph)_2_(H_2_O)_2_∙2H_2_O

Yield: 72%. M.p.: 213–215 °C. Molar cond. (10^−3^ M in DMF): 11.43 ohm^−1^ cm^2^ mol^−1^. µ_eff_(B.M.): 2.94. UV-VIS (DMF) λ_max_ nm: 232 (π→π*), 309 (n→π*), 678, 808 (d→d). IR (KBr, cm^−1^): 3487 ν(N–H), 3393 ν(O–H), 2935 ν(C–H), 1619 ν(C=N), 845 ν(H_2_O), 639 ν(M-N), 498 ν(M–O). Elemental Analysis for C_46_H_46_N_8_O_6_Ni (%): found C 63.80, H 5.79, N 12.18; calculated C 63.81, H 5.36, N 12.95, Mol. mass (g/mol): 865.05.

#### 3.3.12. Bis(4-benzoyl-3-methyl-1-phenyl-2-pyrazolin-5-one phenylhydrazone) Diaquacopper(Ii) Dihydrate Cu(Bmpp-Ph)_2_(H_2_O)_2_∙H_2_O

Yield: 80%. M.p.: 195−197 °C. Molar cond. (10^−3^ M in DMF): 5.98 ohm^−1^ cm^2^ mol^−1^. µ_eff_(B.M.): 1.94.UV-VIS (DMF) λ_max_ nm: 215 (π→π*), 304, 317 (n→π*),516, (d→d). IR (KBr, cm^−1^): 3483 ν(N–H), 3378 ν(O‒H), 2923 ν(C–H), 1618 ν(C=N), 848 ν(H_2_O), 627 ν(M–N), 482 ν(M–O). Elemental analysis for C_46_H_46_N_8_O_6_Cu (%): found C 64.76, H 5.02, N 13.13; calculated C 64.80, H 5.21, N 13.15, Mol. mass: (g/mol): 851.89.

#### 3.3.13. Bis(4-acetyl-3-methyl-1-phenyl-2-pyrazolin-5-one phenylhydrazone) Diaquacobalt(Ii) Dihydrate Co(Ampp-Ph)_2_(H_2_O)_2_∙2H_2_O

Yield: 75%. M.p.: 160−162 °C. Molar cond. (10^−3^ M in DMF): 6.78 ohm^−1^ cm^2^ mol^−1^. µ_eff_(B.M.): 4.49.UV-VIS (DMF) λmax nm: 223 (π→π*), 313, 324 (n→π*), 390, 536, 600 (d→d). IR (KBr, cm^−1^): 3485 ν(N–H), 3391 ν(O‒H), 2923 ν(C–H), 1619 ν(C=N), 844 ν(H_2_O), 621 ν(M–N), 489 ν(M–O). Elemental analysis for C_36_H_42_N_8_O_6_Co (%): found C 57.95, H 5.63, N 15.40; calculated C 58.28, H 5.71, N 15.11, Mol. mass (g/mol): 741.25.

#### 3.3.14. Bis(4-acetyl-3-methyl-1-phenyl-2-pyrazolin-5-one phenylhydrazone) Diaquacopper(Ii) Cu(Ampp-Ph)_2_(H_2_O)_2_

Yield: 79%. M.p.: 212−213 °C. Molar cond. (10^−3^ M in DMF): 12.05 ohm^−1^ cm^2^ mol^−1^. µ_eff_(B.M.): 1.93. UV-VIS (DMF) λ_max_ nm: 217 (π→π*), 310, 323 (n→π*), 401, 594 (d→d). IR (KBr, cm^−1^): 3493 ν(N–H), 3399 ν(O–H), 2926 ν(C–H), 1618 ν(C=N), 821 ν(H_2_O), 628 ν(M–N), 478 ν(M–O). Elemental Analysis for C_36_H_38_N_8_O_4_Cu (%): found C 60.49, H 5.11, N 15.40; calculated C 60.86, H 5.40, N 15.78, Mol. mass (g/mol): 709.85.

### 3.4. X-ray Diffraction Study of Ampp-Ph

Single crystal X-ray diffraction studies were performed at 200 K using a Bruker Kappa Apex II diffractometer with graphite monochromator, Mo Kα radiation (λ = 0.71073 Å). Data collection was carried out with the help of the APEXII while SAINT was used for cell refinement and data reduction [46]. The structure was solved using SHELXS-2014 [47] and refined by least-squares procedures using SHELXL-2014 [47] with SHELXLE [48] as a graphical interface. All non-hydrogen atoms were refined anisotropically. Carbon-bound H atoms were placed in calculated positions and were included in the refinement in the riding model approximation, with *U*_iso_(H) set to 1.2*U*_eq_(C). The H atoms of the methyl groups were allowed to rotate with a fixed angle around the C–C bonds to best fit the experimental electron density (HFIX 137 in the SHELX program suite [47]), with *U*_iso_(H) set to 1.5*U*_eq_(C). Nitrogen-bound H atoms were located on a difference Fourier map and refined freely. The data were corrected for absorption effects by the numerical method using SADABS [46]. Crystallographic data for 4-acetyl-3-methyl-1-phenyl-2-pyrazoline-5-one phenylhydrazone Ampp-Ph reported herein has been deposited with the Cambridge Crystallographic Data Centre, CCDC #. 1417242. CCDC 1417242 contains the supplementary crystallographic data which can be obtained free of charge via http://www.ccdc.cam.ac.uk/conts/retrieving.html, or from the Cambridge Crystallographic Data Centre, 12 Union Road, Cambridge CB2 IEZ, UK; Fax: +44-1223-336-033; E-mail: deposit@ccdc.cam.ac.uk.

### 3.5. Antibacterial Studies

Synthesized compounds were screened against Gram-positive *Staphylococcus aureus*, *Bacillus pumilus*, and Gram-negative *Proteus vulgaris*, *Aeromonas hydrophila* bacterial isolates using the Kirby-Bauer disc diffusion technique at a concentration of 40 mg/mL in DMSO. The antibacterial effect was taken according to the size of inhibition zones around the paper discs with slight modifications to suit experimental conditions [49]. The bacterial isolates were grown in freshly-prepared nutrient broth growth media of suitable antibacterial screening purposes. To a solidified 20 mL Mueller-Hinton agar in inoculation plates, 0.1 mL of test bacteria was spread over the medium using a sterilized spreader. Pre-sterilized filter paper disc with a diameter of 6 mm which have been impregnated into prepared solutions of synthesized compounds were placed in the plates ensuring a reasonable equidistance from each other to give room to avoid zones of inhibition reading interferences. A filter paper disc treated with DMSO was used as control while antibacterial chloramphenicol served as the standard drug and was used as a reference to evaluate the potency of the tested compounds maintaining the same experimental conditions. The plates were left for a few hours in the refrigerator for pre-diffusion and finally transferred to an incubator at 37 °C for 24 h. This procedure was performed in triplicates. The antibacterial activity was determined by measuring the diameter of the zones showing complete inhibition of bacterial growth across the filter paper disc in millimeters, subtracting the diameter of the filter paper disc, and finally dividing by 2 to obtain the exact zone of inhibition. The values were calculated as mean value of triplicates.

### 3.6. Antioxidant (Free Radical Scavenging) Activity

Free radical scavenging properties of the synthesized compounds were tested against the free radical of 1,1-diphenyl-2-picryl-hydrazil (DPPH), employing the method of Blois with some modifications [50]. A 0.1 mmol solution of the DPPH in methanol was prepared, and 1 mL of the solution was added to 3 mL prepared solutions of the test compounds in a mixture of DMSO and methanol in a mole ratio of 1:9, respectively, at different concentrations (0.13, 0.25, and 0.50 mg/mL). The same procedure was carried out for ascorbic acid (reference drug) and an equal volume of dissolving solvents as control. The mixture was shaken vigorously and allowed to stand at room temperature in the dark for 30 min. With the use of a spectrophotometer at a wavelength of 517 nm the absorbance of each solution was measured. The capabilities of synthesized compounds to scavenge DPPH radical were calculated using the expression;

$$\mathrm{Scaveging activity}(\%)=\frac{A_{0}-A_{1}}{A_{0}}\times 100$$

where A_0_ is the absorbance with control sample and A_1_ is the absorbance with test samples including that of the standard drug.

## 4. Conclusions

A new set of acylpyrazolone phenylhydrazones, their transition metal complexes inclusive, have been successfully prepared, adequately identified, and contributed to the pull of compounds with unique properties that are potential antimicrobial agents, which may become an alternative replacement for disease-resistant therapeutic agents. The structural elucidation, as well as proposed molecular structures of these ligands based on analytical, NMR, and mass spectroscopy results, is in agreement with the single crystal structure of their 4-benzoyl-3-methyl-1-phenyl-2-pyrazolin-5-one phenylhydrazone reported earlier. Their metal complexes have mostly exhibited unprecedented electronic spectra associated with proposed transitions, but they have shown expected magnetic properties which have been used alongside their FTIR and thermogravimetric analysis (TGA) results to justify their proposed structures. The compounds have shown generally low antibacterial properties, but good antioxidant activity, with the metal complexes having a better bioactivity overall.

## Figures and Tables

**Table 1:** Crystal data for Ampp-Ph.

Compound	Ampp-Ph
Formula	C_18_H_18_N_4_O
Crystal colour and form	Golden yellow/Block
Formula weight	306.36
Crystal system	Triclinic
Space group	P-1
*a*	8.7006(5) (Å)
*b*	9.6088(5) (Å)
*c*	9.9124(5) (Å)
*α*	104.740(2)°
β	96.360(2)°
γ	103.196(2)°
*V*	767.62(7) (Å^3^)
*Z*	2
*D*_(calc)_	1.326 (Mg cm^−1^)
*F*(000)	324
θ range	2.2–28.3 (°)
Crystal size	0.35 × 0.47 × 0.53 (mm)
Total Reflection measured	20,159
*R*	0.0378
*wR_2_*	0.1078
*S*	1.02
Independent/observed	3801/3374
Mu(MoKa)	0.71073 (/mm)
Temperature	200 (K)
Parameters	218

**Table 2:** Zone of growth inhibition exhibited by phenylhydrazone and metal complexes at 40 mg/mL (mm).

Ligand and Complexes	*Staphylococus aureus*	*Bacillus pumillus*	*Proteus vulgaris*	*Aeromonas hydrophillia*
Bmpp-Dh	4.0	8.3	NI	NI
Ampp-Dh	24.0	9.5	NI	4.5
Bmpp-Ph	12.0	10.0	8.0	NI
Mn(Bmpp-Ph)_2_(H_2_O)_2_∙2H_2_O	10.0	NI	8.0	NI
Co(Bmpp-Ph)_2_(H_2_O)_2_∙H_2_O	8.0	12.0	NI	NI
Ni(Bmpp-Ph)_2_(H_2_O)_2_∙2H_2_O	NI	8.0	4.0	10.0
Cu(Bmpp-Ph)_2_(H_2_O)_2_∙H_2_O	12.0	6.0	8.0	NI
Mn(Bmpp-Dh)_2_(H_2_O)_2_	8.0	7.0	NI	NI
Co(Bmpp-Dh)_2_(H_2_O)_2_∙H_2_O	8.3	7.0	NI	NI
Ni(Bmpp-Dh)_2_(H_2_O)_2_∙H_2_O	10.5	6.0	12.0	12.0
Cu(Bmpp-Dh)_2_(H_2_O)_2_∙2H_2_O	NI	8.5	NI	12.0
Co(Ampp-Ph)_2_(H_2_O)_2_∙2H_2_O	8.0	12.0	NI	6.0
Cu(Ampp-Ph)_2_(H_2_O)_2_	4.0	4.0	NI	NI
Mn(Ampp-Dh)_2_(H_2_O)_2_∙H_2_O	8.0	8.5	9.5	5.0
Co(Ampp-Dh)_2_(H_2_O)_2_	15.5	4.0	NI	20.0
Ni(Ampp-Dh)_2_(H_2_O)_2_	8.0	12.5	NI	8.3
Cu(Ampp-Dh)_2_(H_2_O)_2_	13.0	12.3	NI	20
Chloramphenicol	30.0	20.0	42.0	40.0
DMSO	NI	NI	NI	NI

NI = no inhibition.

**Table 3:** Antioxidant scavenging activity data of 2,4-dinitrophenylhydrazones and their metal complexes (%).

Ligand and Complexes	Percentage Antioxidant Activity		
	0.50 mg/mL	0.25 mg/mL	0.13 mg/mL
Bmpp-Dh 1	‒	31.05	30.70
Ampp-Dh 2	‒	63.27	76.33
Bmpp-Ph 3	62.48	89.11	87.79
Mn(Bmpp-Ph)_2_(H_2_O)_2_∙2H_2_O 3a	52.42	42.76	64.61
Co(Bmpp-Ph)_2_(H_2_O)_2_∙H_2_O 3b	17.22	3.13	32.32
Ni(Bmpp-Ph)_2_(H_2_O)_2_∙2H_2_O 3c	37.33	4.17	40.94
Cu(Bmpp-Ph)_2_(H_2_O)_2_∙H_2_O 3d	‒	12.98	1.73
Mn(Bmpp-Dh)_2_(H_2_O)_2_ 1a	‒	68.83	52.28
Co(Bmpp-Dh)_2_(H_2_O)_2_∙H_2_O 1b	8.58	59.56	47.11
Ni(Bmpp-Dh)_2_(H_2_O)_2_∙H_2_O 1c	87.92	62.80	61.28
Cu(Bmpp-Dh)_2_(H_2_O)_2_∙2H_2_O 1d	2.28	89.90	53.76
Co(Ampp-Ph)_2_(H_2_O)_2_∙2H_2_O 4a	60.07	87.25	80.64
Cu(Ampp-Ph)_2_(H_2_O)_2_ 4b	38.35	57.36	44.76
Mn(Ampp-Dh)_2_(H_2_O)_2_∙H_2_O 2a	68.48	85.86	87.42
Co(Ampp-Dh)_2_(H_2_O)_2_ 2b	‒	55.50	55.98
Ni(Ampp-Dh)_2_(H_2_O)_2_ 2c	65.32	84.01	84.83
Cu(Ampp-Dh)_2_(H_2_O)_2_ 2d	35.73	85.40	82.98
Ascorbic acid std	87.62	93.63	91.99

## Abbreviations

DMF	Dimethylformamide
DTG	Thermogravimetric derivative
DPPH	1,1-diphenyl-2-picryl-hydrazil
DMSO	Dimethylsulfoxide
TLC	Thin Layer Chromatography
